# Solving a 50 year mystery of a missing *OPA1 *mutation: more insights from the first family diagnosed with autosomal dominant optic atrophy

**DOI:** 10.1186/1750-1326-5-25

**Published:** 2010-06-14

**Authors:** Nico Fuhrmann, Simone Schimpf, York Kamenisch, Beate Leo-Kottler, Christiane Alexander, Georg Auburger, Eberhart Zrenner, Bernd Wissinger, Marcel V Alavi

**Affiliations:** 1 Molecular Genetics Laboratory, Institute for Ophthalmic Research, Centre for Ophthalmology, University of Tuebingen, Germany; 2 Department of Dermatology, Eberhard Karls University Tuebingen, Germany; 3 Centre for Ophthalmology, Institute for Ophthalmic Research, University of Tuebingen, Germany; 4 Department of Neurodegeneration, Max-Delbrück-Center for Molecular Medicine, Berlin, Germany; 5 Section Molecular Neurogenetics, Department of Neurology, Goethe University, Frankfurt am Main, Germany

## Abstract

**Background:**

Up to the 1950s, there was an ongoing debate about the diversity of hereditary optic neuropathies, in particular as to whether all inherited optic atrophies can be ascribed to Leber's hereditary optic neuropathy (LHON) or represent different disease entities. In 1954 W. Jaeger published a detailed clinical and genealogical investigation of a large family with explicit autosomal dominant segregation of optic atrophy thus proving the existence of a discrete disease different from LHON, which is nowadays known as autosomal dominant optic atrophy (ADOA). Since the year 2000 ADOA is associated with genomic mutations in the *OPA1 *gene, which codes for a protein that is imported into mitochondria where it is required for mitochondrial fusion. Interestingly enough, the underlying mutation in this family has not been identified since then.

**Results:**

We have reinvestigated this family with the aim to identify the mutation and to further clarify the underlying pathomechanism. Patients showed a classical non-syndromic ADOA. The long term deterioration in vision in the two teenagers examined 50 years later is of particular note 5/20 to 6/120. Multiplex ligation probe amplification revealed a duplication of the *OPA1 *exons 7-9 which was confirmed by *long distance PCR *and cDNA analysis, resulting in an in-frame duplication of 102 amino acids. Segregation was verified in 53 available members of the updated pedigree and a penetrance of 88% was calculated. Fibroblast cultures from skin biopsies were established to assess the mitochondrial network integrity and to qualitatively and quantitatively study the consequences of the mutation on transcript and protein level. Fibroblast cultures demonstrated a fragmented mitochondrial network. Processing of the OPA1 protein was altered. There was no correlation of the *OPA1 *transcript levels and the OPA1 protein levels in the fibroblasts. Intriguingly an overall decrease of mitochondrial proteins was observed in patients' fibroblasts, while the *OPA1 *transcript levels were elevated.

**Conclusions:**

The thorough study of this family provides a detailed clinical picture accompanied by a molecular investigation of patients' fibroblasts. Our data show a classic *OPA1*-associated non-syndromic ADOA segregating in this family. Cell biological findings suggest that OPA1 is regulated by post-translational mechanisms and we would like to hypothesize that loss of OPA1 function might lead to impaired mitochondrial quality control. With the clinical, genetic and cell biological characterisation of a family described already more than 50 years ago, we span more than half a century of research in optic neuropathies.

## Background

During the first half of the last century, there was a controversial debate about the clinical and etiological unity of hereditary optic neuropathies. Some ophthalmologists favored the idea that the majority of cases are part of the manifestation spectrum of the Optic Atrophy described by Theodor Leber [1], that we now know as Leber's hereditary optic neuropathy (LHON) [2-4]. Others argued that there are different disease entities and suggested discriminating different forms of optic atrophy [5-7]. In 1954, Wolfgang Jaeger reported his genealogical and clinical findings in an extended German family spanning five generations. In this pedigree he could clearly demonstrate that optic atrophy is inherited as a dominant trait including male-to-male transmission. In addition, he pointed out the presence of blue-yellow color vision disturbances in the affected subjects in this family that contrasts to the red-green defect typically present in families with LHON [8]. These features, together with a thorough review of prior clinical reports, enabled him to establish autosomal dominant optic atrophy (ADOA) as a distinct disease entity.

Nowadays, ADOA is a well established disease entity and considered the most frequent hereditary optic atrophy besides LHON. ADOA is clinically characterized by a juvenile onset with a progressive, bilateral reduction of visual acuity, a cecocentral scotoma, temporal pallor of the optic disc and tritanopia as the most typical type of color vision defect [9,10]. There is considerable intra- and interfamilial variability in progression of the disease as well as in the severity of the visual impairments, ranging from very mildly affected subjects to legally blind patients [11-13]. Moreover, asymptomatic carriers have been reported in many pedigrees demonstrating reduced penetrance [14-16]. Histopathologic investigations have shown a loss of retinal ganglion cells (RGCs) and thinning of the nerve fiber layer [17,18] which later could be confirmed in an ADOA mouse model [19]. A major locus for ADOA was mapped to chromosome 3q28-3q29 by linkage analysis [20] and subsequently the disease-causing gene *OPA1 *was identified by our group and an independent group [21,22]. Besides *OPA1*, two further gene loci for ADOA, *OPA4 *[23] and *OPA5 *[24] have been mapped but the underlying genes remain unknown so far. In addition, a small number of families have been reported with *OPA3 *mutations that cause ADOA associated with early onset cataract [25]. Given the large number of reports of families with *OPA1 *mutations, the other loci seem to play only a minor role. There are more than 200 *OPA1 *mutations listed in the eOPA1 database http://lbbma.univ-angers.fr/eOPA1[26]. Most of these mutations have been identified by conventional, qualitative genetic screening techniques that are insensitive for genomic alterations (i.e. gross deletions or duplications). Only one family with a complete *OPA1 *gene deletion has been reported in the literature [27] and just recently we have been able to demonstrate that genomic rearrangements may constitute a considerable proportion of causal *OPA1 *gene mutations [28].

The OPA1 protein is a nuclear encoded, dynamin-related GTPase which is imported into mitochondria and anchored to the inner membrane of the organelle [29]. Together with mitofusin 1 and 2 it plays a major role in mitochondrial fusion and therefore is important for the maintenance of the mitochondrial network morphology and dynamics [30,31]. Furthermore, OPA1 has been linked to mitochondrial cristae stability and its remodeling in apoptosis [32-34]. How specific OPA1 mutants affect these principal functions has only been sparsely investigated and why mutations in *OPA1 *cause a mostly selective degeneration of RGCs is unknown. On the other hand there are case reports of syndromic forms of ADOA that include extra-ocular neurological features (sensorineural deafness, ataxia, axonal sensory motor-polyneuropathy, chronic progressive external ophthalmoplegia, mitochondrial myopathy) which have been associated with multiple mitochondrial DNA (mtDNA) deletions [35,36]. However, mtDNA deletions can not only be found in muscles from patients with syndromic ADOA, but seem to be present also in muscle biopsies from patients with non-syndromic ADOA [37].

Here we report the identification and re-evaluation of the large family described by W. Jaeger in 1954. Using copy-number sensitive techniques, we have been able to finally identify the disease causing mutation in this family. We provide an update of the pedigree, a clinical follow up of a branch of this family as well as a qualitative and quantitative investigation of mutated *OPA1 *transcripts and OPA1 protein in fibroblast cell lines established from two patients of this family.

## Results

**Figure 1:**
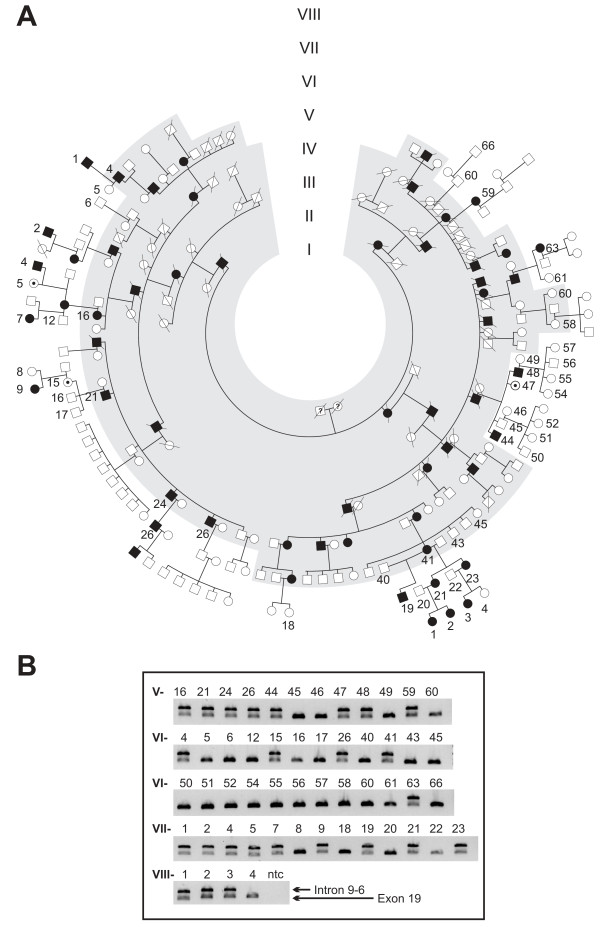
**Pedigree of the investigated ADOA family and segregation analysis of the *OPA1 *exon 7-9 duplication**. **(A) **Updated pedigree of the family. The gray area indicates the original pedigree as described by W. Jaeger 1954. Only persons of whom DNA samples were available are indexed. **(B) **Segregation analysis applying a PCR assay specific for the OPA1 exon 7-9 duplication. A fragment covering exon 19 sequences was co-amplified as internal control. Subjects are numbered according to the pedigree. Ntc - non template control.

The original ADOA family described in 1954 was re-contacted with the help of Prof. W. Jaeger (now deceased) and most of the blood samples and medical records were collected in a field study in the mid-90s. The pedigree was updated through several personal interviews with family members, the latest in 2008 (Figure 1). The reconstructed pedigree now comprises a total of 216 family members of whom 57 are affected or were reported to have been affected. In comparison with the pedigree reported in 1954 we noted 19 additional family members affected by ADOA. DNA samples of 22 affected and 31 unaffected family members were collected for this study and fibroblast cultures for functional investigations were established from skin biopsies of two affected subjects (Figure 1, V-24 and V-26).

### Long-term follow-up of patients revealed a typical non-syndromic ADOA

**Figure 2:**
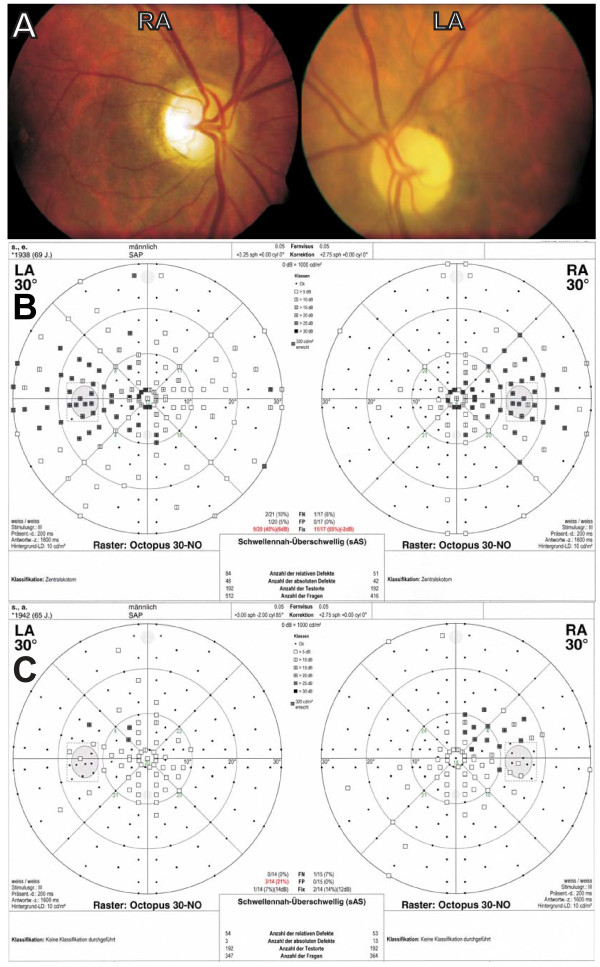
**Clinical picture of two patients that had been examined already by W. Jäger**. **(A) **Funduscopy of patient B (V-26) showed pallor of the optic disc with temporal prominence. **(B) **Visual field of patient A (V-24) showed a clear central scotoma. **(C) **Patient B showed a decentered central scotoma (excentric retinal locus of fixation, note conjugate displacement of the blind spot).

Two affected brothers who were already described in the original report underwent a full ophthalmological re-examination more than 50 years after their initial clinical description (Figure 1A, V-24 and V-26). Patient A (V-24), now aged 69 years and patient B (V-26), now 66 years old, were first examined at the age of 15 and the age of 11, respectively. W. Jaeger reported that both complained of reduced vision in school and already experienced modest loss of visual acuity at this age. Since then visual acuity dropped in both patients from 5/25 and 5/20, respectively, to each 6/120 in both eyes. A slow course of disease progression was also confirmed by self-reports. Neither of them remembered periods of fast loss of visual acuity. Yet both patients have now developed cecocentral scotoma and an optic atrophy progressed to a complete pallor of the optic nerve head, especially of the temporal part of the disc (Figure 2). In comparison with the results of former color vision tests described in Jaeger's original paper, both patients reported further deterioration of their color vision. Using Ishihara's test charts they both could formerly identify at least 5 of the test plates. At the recent examination both patients did not see even the reference plate of the test. Using Stilling-Hertel test charts, patient A could previously identify 11, patient B 13 of the plates. At the recent examination patient A could only identify 2 and patient B 7 plates. Over the period of 54 years, the patients' ability to discriminate between colors has severely diminished.

In addition patient A suffered from glaucoma; the intraocular pressure (IOP) was 21/25 mmHG (R/L) without medication, while IOP was still in the normal range in patient B (R/L 18/19 mmHG). Patient B received a stent in 2006. Otherwise the medical history was unremarkable and neither of the patients had diabetes mellitus or hearing impairments, typical symptoms in syndromic forms of optic atrophy. In conclusion both patients show a clear and classical non-syndromic ADOA.

### Mutation analysis showed a duplication of exons 7 to 9 in the *OPA1 *gene

**Figure 3:**
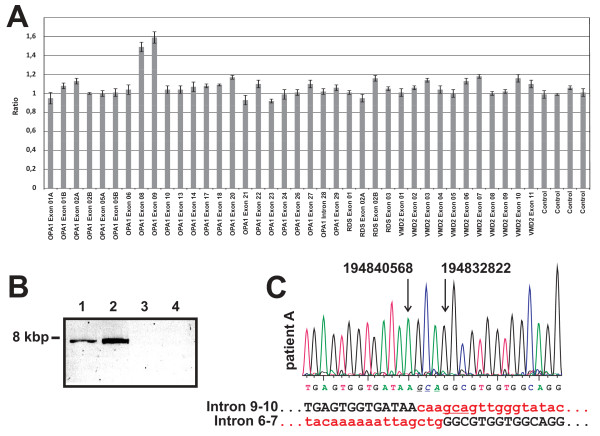
**Identification and characterization of the exon 7-9 duplication in *OPA1 *by MLPA**. **(A) **Typical read out of the MLPA experiment of index patient (VIII-4) depicting a significant increase for the exon 8 and 9 probes. **(B) **Long distance PCR amplification with forward and reverse primer located both in exon 8 yielded a fragment of approximately 8 kb in affected family members only (line 1: patient A, line 2: patient B, line 3: not affected control, line 4: no template control). **(C) **Sequencing of the amplicon shown in (B) reveals the genomic breakpoint and demonstrates that exon 7 is duplicated as well. The numbers above the arrows give the genomic position (human genome assembly, build 36.3), black capital letters indicate retained sequences of intron 9-10 and intron 6-7, respectively, while small red letters show the deleted sequence portion. The underlined 3 nucleotides can be found as reminiscence of the deletion event.

An initial microsatellite marker analysis revealed significant linkage with the *OPA1 *locus (C. Alexander and G. Auburger, unpublished results). However, subsequent screening of the *OPA1 *gene by means of DNA sequencing of the coding exons and flanking intron sequences from PCR-amplified genomic DNA did not reveal any putative pathogenic mutation. We therefore applied Multiplex Ligation Probe Amplification (MLPA) to analyse for copy number variations and genomic rearrangements. Indeed, MLPA showed a significant signal increase for the *OPA1 *exon 8 and 9 probe amplicons in the index patient in three independent experiments (Figure 3A). Using oligonucleotides placed both - forward and reverse - in exon 8 for a *long distance PCR *we were able to amplify a specific PCR product of approximately 8 kb in samples of affected individuals only (Figure 3B). Primer walking revealed the presence of a recombined sequence that fuses the 5' part of intron 9 (breakpoint at np194840568, human genome assembly march 2006) with the 3' part of intron 6 (np194832822) of the *OPA1 *gene (Figure 3C). From this we conclude that exons 7-9 plus the flanking intron sequences are duplicated in this family. This mutation was also found in two other ADOA families [28]. *In silico *analysis revealed Type I transposon elements adjacent to both breakpoints (not shown). We developed a PCR assay that is specific for this duplication and performed a segregation analysis in all family members of whom a DNA sample was available (Figure 1B). We found that all affected family members carry the duplication. In addition, three asymptomatic family members (Figure 1B; V-47, VI-15, VII-5) were mutation carriers. Taking into account that 22 of the 25 mutation carriers are affected we calculate a penetrance of 88% in this family.

### The mitochondrial network is fragmented in patients' fibroblasts

**Figure 4:**
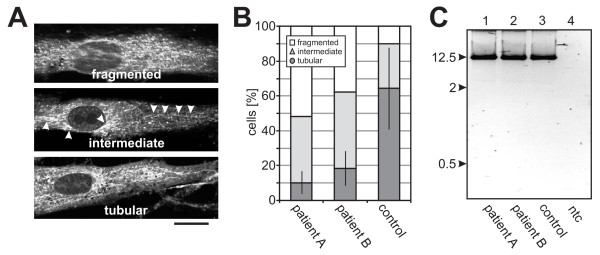
**Assessment of the mitochondrial network morphology**. **(A) **Representative micrographs of cells with different mitochondrial network morphology. The different categories are indicated. Arrow heads accentuate tubular structures in the mitochondrial network classified as "intermediate". Scale bar, 20 μm. **(B) **Cells have been classified by two independent qualified persons in a blinded experiment with a third person taking the micrographs (n > 100 for each group). The number of cells that exhibit tubular mitochondrial network morphology is reduced in patients' fibroblasts compared to a control. (**C**) Amplification of a 12.5 kb mtDNA fragment did not reveal any specific mtDNA deletions for patients or controls. Ntc - non template control.

Since OPA1 is necessary for mitochondrial fusion [30,31], we morphometrically assessed the mitochondrial network in fibroblasts of the two patients after mitotracker staining and confocal imaging. In comparison with wild type control cells we found a significantly decreased proportion of cells with a tubular mitochondrial network when cultured in standard glucose medium (Figure 4A &4B). Replacement of glucose by galactose in the growth medium to avoid glycolysis reinforced the decrease in cells with tubular mitochondrial network (not shown). These results demonstrate that mutant cells are clearly impaired in their ability to fuse mitochondria. We tested also the integrity of the mtDNA in these cell lines by the amplification of a 12.5 kb fragment, but did not notice any differences from controls (Figure 4C).

### Qualitative and quantitative cDNA analyses revealed an in-frame duplication, a reduced expression level of mutant transcripts and an overall increase of *OPA1 *transcripts in fibroblasts

**Figure 5:**
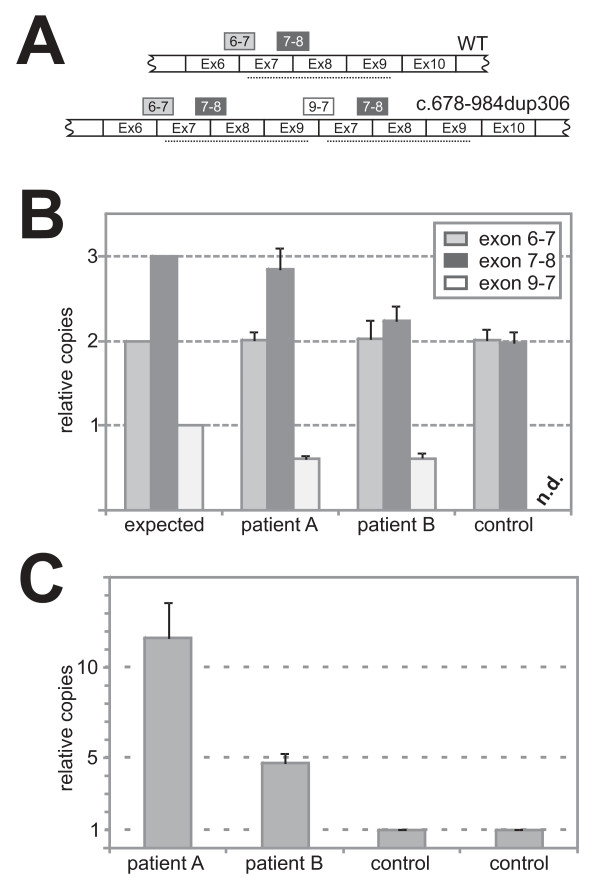
**Quantification of the different *OPA1 *transcripts derived from the mutant and the WT allele**. **(A) **A clarifying scheme about the strategy for the relative quantification with boxes indicating the different amplicons of exon/exon junctions and their relative ratios if one assumes that both transcripts are equally abundant. **(B) **One would expect a ratio of exon 7-8 to exon 6-7 to exon 9-7 of 3 to 2 to 1 when transcripts of both alleles are equally abundant (expected); patients clearly presented with reduced levels of exon 7-8 and 9-7 amplicons indicating an imbalance in favor of the transcripts derived from the WT allele (patient A & patient B); controls show no difference in the quantification of the exon 7-8 and 6-7 amplicons, proving that the method works very accurate. **(C) **The readout of a typical quantification of the exon 6-7 junction in relation to GAPDH shows a significant up regulation of *OPA1 *in patients' fibroblasts.

To study the consequences of this mutation we analysed cDNA from patients' fibroblast cell lines. Thereby we found minor fragments that may represent aberrantly spliced mutant transcripts. Subcloning and sequencing of the major fragment of increased length revealed that this cDNA species had the additional exons correctly integrated as a duplication of 306 bp (c.678-984dup306). This duplication results in an in-frame duplication of 102 amino acids (p.L227_K328dup102). To test if there is an imbalance between the transcripts of the mutated and the WT allele in our patients we quantified allelic transcript levels by pyrosequencing as described previously [38]. Analyses of the *OPA1 *exon 21 polymorphism (c.2109C > T) revealed a mean percentage ratio for the two alleles of 48.7% to 51.3% (S.D. = 1.2). This shows that both alleles are transcribed similar. We also quantified different *OPA1 *exon-exon junctions in relation to *GAPDH *by real time PCR to verify this result. If both alleles are transcribed similarly one would expect a ratio of exon 9-7 (x) to exon 6-7 (y) to exon 7-8 (z) of one-to-two-to-three (x : y : z = 1 : 2 : 3; see Figure 5A &5B, line 1 for an overview). However, we found a ratio x : y : z of 0.60 (S.D. = 0.05) : 2 : 2.85 (S.D. = 0.25) in patient A and a ratio x : y : z of 0.62 (S.D. = 0.07) : 2 : 2.24 (S.D. = 0.18) for patient B, respectively (Figure 5B, line 2 and 3). Controls displayed a ratio y : z of 2 : 1.98 (S.D. = 0.14) thus proving the reliability of this method (Figure 5B, lane 4). This inconsistency in the results of both assays can be interpreted in a way that a fraction of the transcripts derived from the mutant allele may be spliced correctly (i.e. eliminating the 3 duplicated exons and presenting as wild type) or spliced aberrantly, or both. Of note, the overall steady-state levels of *OPA1 *transcripts in patients' fibroblasts was more than two-fold increased in comparison to the control fibroblasts (Figure 5C).

### Qualitative and quantitative protein analysis reveals reduced levels of OPA1 and altered processing of the different OPA1 isoforms

**Figure 6:**
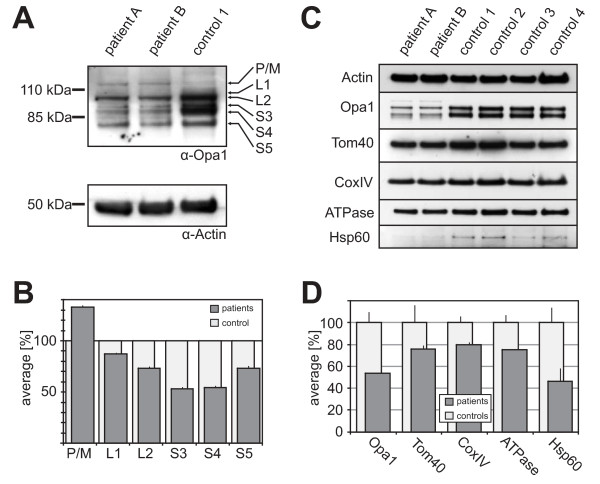
**Reduced OPA1 protein amount and altered processing in fibroblasts from patients with ADOA**. **(A) **Quantitative western blot against OPA1 (upper panel) and actin (lower panel) of the indicated probes. Arrows indicate the corresponding bands; P/M: precursor/mutant protein, L1 to S5 bands according to Duvezin-Caubet et al. 2007 [39]. **(B) **Densitometric evaluation of the different OPA1 isoforms from the western blot shown in A as mean ± S.D. from patients vs. control. OPA1 protein levels are reduced in the patients' cell lines and the unequal reduction of the different corresponding OPA1 isoforms suggest altered proteolytic cleavage of OPA1. **(C) **Quantitative western blot against indicated mitochondrial proteins in total cell lysates of the indicated probes. **(D) **Densitometric quantification of the western blot shown in C as mean ± S.D. from patients vs. controls. The analysis shows that all tested mitochondrial proteins are less abundant in patients' cell lines compared to controls.

For a qualitative and quantitative analysis of OPA1 protein we used total cell lysates from fibroblast cell lines of patients V-24 and V-26 and four control fibroblast cell lines derived from healthy individuals. The duplicated exons 7-9 of the mutant *OPA1 *allele result in an in-frame duplication of 102 amino acid residues within the GTPase domain of the OPA1 protein (p.L227_K328dup102), that is expected to increase its molecular weight by 11.8 kDa. We consistently found a reduction of all OPA1 protein isoforms in both patients' cell lines in relation to actin (Figure 6A). The different OPA1 isoforms were assigned according to their size as described before [39]. Still we are aware that the assignment is rather hypothetical and additional experiments would be necessary to define precisely the single bands. However, according to its size the P/M band could represent either OPA1 precursor or mutant protein or both. Densitometric analysis of the different isoforms revealed that the S3 and S4 isoforms were reduced to approx. 50% of their levels in controls while L1, L2 and S5 were reduced not that strong (Figure 6B). The overall OPA1 protein level (excluding the precursor/mutant form) was reduced to approx. 66%. The observed changes in the relative abundance of the different OPA1 isoforms implicate altered proteolytic processing of OPA1 in mitochondria (e.g. reduced processing when L1 and S3 constitutes splice variant 7 and L2, S4 and S5 constitute splice variants 1 and 5 as suggested before [39,40]).

Since we found a reduction of all OPA1 isoforms in relation to actin, we assessed several other mitochondrial proteins (Tom40, CoxIX, ATPase β, Hsp60) in total cell lysates by westernblot analyses and subsequent densitometric measurements to clarify whether other mitochondrial proteins are reduced in their abundance as well. In comparison to four control fibroblast cell lines we found a reduction of all tested mitochondrial proteins of 20 to 55% consistent in both patients' fibroblast cell lines (Figure 6C &6Ds). This finding shows that together with OPA1 other mitochondrial proteins seem to be reduced as well.

## Discussion

Here we report the identification of a large intragenic duplication in the *OPA1 *gene in a historic family with ADOA that was first described in 1954 and that was decisive for the definition of ADOA as a separate disease entity [8]. The updated pedigree now comprises a total of 216 family members of whom 57 are affected or were reported to suffer from ADOA. The family thus constitutes probably one of the largest reported in the ADOA literature and may enable further investigations of secondary factors that modulate disease expression and penetrance. Based on our genotyping results we calculated a penetrance of 88% in this family. This is in line with a previous thorough study of several Australian families with ADOA that reports also a penetrance of 88% and 82.5% for the fully ascertained sibships, respectively [14,15]. Notably, all unaffected mutation carriers in the family presented herein are females and we previously reported two more unaffected female mutation carriers in other families, one with the same exon 7-9 duplication mutation and one with a complete deletion of the *OPA1 *gene [28]. All of these unaffected mutation carriers have affected siblings or children which share by definition the same mtDNA haplogroup. This shows that the mtDNA background does not play a major role in governing penetrance in ADOA and we would like to speculate instead about an x-chromosomal inherited QTL or environmental factors. In summary, the studied family presents a well-defined clinical picture for non-syndromic ADOA. There is no evidence of associated extra-ocular symptoms, either in the thorough clinical investigations by W. Jaeger or in our recent re-examinations.

In contrast to other inherited blinding diseases, in ADOA the affected gene is expressed ubiquitously [21,41]. With limitations, this offers the possibility to study the consequences of the disease causing mutation on cellular level in any tissue or cell line derived from patients. For the study presented here, we have established two fibroblast cell lines from skin biopsies of the two affected brothers at the age of 69 and 66, respectively and compared them with four different control fibroblast cell lines with the same passage number. Two of the latter were obtained from young children's circumcision specimens whereas the other two derived from skin biopsies of a middle-aged donor and a donor in his late sixties, which served as the primary control. All donors were healthy individuals. Unfortunately we were not able to obtain skin biopsies of the unaffected brothers. However, to our knowledge, donor age has only a minor effect on the replicative lifespan of fibroblasts, in contrast to passage numbers of the cells and/or health status of the donors [42,43], which was both carefully controlled in this study. Donor age shows also only minor effects on mitochondrial enzyme activity [44]. In agreement with this, we didn't find any specific mtDNA deletions in patients' cell lines or control cells that would refer to a premature aging effect of the cells.

Nevertheless, we found a more fragmented and less tubular morphology of the mitochondrial network in the patients' cell lines. Knockout mutants of the *OPA1*-homologue mgm1/msp1 in yeast show also fragmentation of mitochondria and a reduction of mtDNA content [45,46]. A quite similar mitochondrial phenotype could also be observed by down-regulation of *OPA1 *expression by RNA interference in HeLa cells, which caused fragmentation of the mitochondrial network, loss of mitochondrial membrane potential and disorganization of the mitochondrial cristae [33,47]. Spinazzi and colleagues also reported fragmentation of the mitochondrial network in fibroblasts as well as myotubes from patients that harbor a deletion in the GTPase domain of OPA1 (c1410_144314del38) [48].

We found reduced OPA1 protein levels in fibroblasts obtained from patients with non-syndromic ADOA that harbor a 3-exon-duplication in the GTPase domain of OPA1 when compared to controls. This is in line with previous reports on other *OPA1 *mutations in patients with ADOA, including the deletion in the GTPase domain of OPA1 mentioned above, or Opa1 mouse models, which all report reduced OPA1 protein levels, regardless of the underlying *OPA1 *mutation [48-52]. Based on this and the description of different families with non-syndromic ADOA that segregate heterozygous deletions of the complete *OPA1 *gene [27,28], haploinsufficiency is believed to be a major pathomechanism in *OPA1 *associated non-syndromic ADOA.

On the other hand our data show that there might be an accumulation of *OPA1 *transcripts in the patients' cells, which would be in accordance with the observed augmentation of the precursor/mutant protein (P/M band), but in contrast to the reduction of the other OPA1 isoforms in these cells. Previously, we have reported reduced Opa1 protein levels in various tissues obtained from Opa1 mutant mice. In these mice, the *Opa1 *transcript levels were not altered compared to littermate controls [49]. Taken together, there seems to be no correlation of the transcript levels and the protein levels for OPA1, which suggests that OPA1 is regulated on protein levels. This is supported by a study on OPA1 in conjunction with heart failure that also found no correlation of *OPA1 *transcript and OPA1 protein levels [53]. In conclusion, this suggests that this 102 amino acid duplication in the GTPase domain of OPA1 leads to altered OPA1 function rather than haploinsufficiency. Taking into account that loss of one complete OPA1 allele leads to a comparable clinical picture (i.e. non-syndromic ADOA) [27,28], one might speculate that OPA1 function is reduced by this particular mutation. *Then why and how are OPA1 protein levels reduced in ADOA? *The ubiquitine-proteasome pathway is not involved in the degradation of OPA1 as could be shown in Opa1 mutant mice [49].

Recently, it has been proposed that mitochondrial fission and selective fusion serves as *mitochondrial quality control*. Experiments with photo labeled mitochondria revealed that selective fusion separates functional from dysfunctional mitochondria [54-56]. Dysfunctional mitochondria display a reduced membrane potential ΔΨ [56], which results in increased proteolytic cleavage of OPA1 [57-62]. As a consequence dysfunctional mitochondria are not capable to fuse anymore and are degraded by autophagy [54,56]. According to this, a change in OPA1 function which reduces mitochondrial capability to fuse should also result in increased mitochondrial degradation by autophagy. Indeed, increase in autophagy has been observed in the optic nerve of Opa1 mutant mice [63]. This might be an explanation for the discrepancy of *OPA1 *transcript and OPA1 protein levels. In addition, this would be in agreement with the observed fragmented mitochondrial network, the reduced proteolytic processing and the reduction of OPA1 and other mitochondrial proteins in the cell lines from patients with ADOA found in this study. Altered *mitochondrial quality control *would also explain mitochondrial dysfunction and/or the accumulation of mtDNA deletions as described for patients with ADOA [37].

Still it needs to be shown, if loss of OPA1 function impairs mitochondrial function, which leads to more dysfunctional mitochondria and subsequent removal of these dysfunctional mitochondria, or if loss of OPA1 function impairs *mitochondrial quality control *directly, which then leads to increased clearance of apparently functional mitochondria, too.

## Methods

### Patients and Subjects

We included 53 family members in this study who have been recruited in a field study in the mid-nineties. An update of the pedigree was obtained from recent personal interviews with various family members. Two of the patients underwent a full ophthalmological examination at the University Ophthalmic Hospital in Tuebingen in 2008. Informed consent was obtained for blood samples and skin biopsies. The study was performed in accordance to the tenets of the Declaration of Helsinki and was approved by the local ethics committee.

### Cell culture and staining of the mitochondrial network

Fibroblast cell lines were established from skin biopsies from patients and compared to 4 different healthy control cell lines with the same passage number. All cell lines were free of mycoplasma contaminations. Cells were propagated in minimal essential medium supplemented with 10% fetal calf serum (FCS) and antibiotics under standard conditions. For assessment of the mitochondrial network structure, cells were cultured for 24 hours in glucose-free DMEM supplemented with either 10% FCS and 0.45% (w/v) glucose or 10% FCS, 5 mM galactose and 5 mM pyruvate prior to staining with 10 nM Mitotracker for 45 min at 37°C in the corresponding medium. The morphology of the mitochondrial network was qualitatively assessed by two independent people who did not know either the condition or the origin of the pictures which had been taken by a third person with a fluorescence microscope (AXIO Imager Z1 with ApopTome, Zeiss, Germany).

### Isolation of DNA and RNA

Genomic DNA was extracted from venous blood samples applying standard salting-out procedure, or from cultivated fibroblasts applying High Pure PCR Template Preparation Kit (*Roche*, Mannheim, Germany). Total RNA was isolated from whole blood drawn in PAXgene tubes using the PAXgene blood RNA kit (*Qiagen*, Hilden, Germany). RNA from fibroblasts was obtained using the RNeasy Kit (*Qiagen*) and subsequently treated with DNAse (*New England Biolabs*, Frankfurt, Germany). RNA was reverse transcribed using either the Long Range 2 Step RT-PCR Kit (*Qiagen*) or the Super Script III First-Strand Synthesis System for RT-PCR (*Invitrogen*, Carlsbad, USA) with oligo-dT-primers or random hexamers.

### Multiplex ligation-dependent probe amplification (MLPA)

MLPA reactions were performed using the P97 and P229 kit from *MRC-Holland *(Amsterdam, The Netherlands) following the manufacturers' instructions and applying an exactly defined amount of sample DNA (100 ng). Reactions were performed in triplicates and compared with parallel processed controls. The amplified MLPA products were separated on a 3100 Capillary Sequencer (*Applied Biosystems*, Darmstadt, Germany) and analysed using the Coffalyser spreadsheet (control probe analysis, *MRC-Holland*).

### Breakpoint identification and segregation analysis

The exon 7-9 duplication was confirmed by long distance PCR, carried out with the TaKaRa LA Taq™ kit (*TAKARA BIO INC*., Shiga, Japan) applying both forward and reverse oligonucleotides placed in Exon 8, Exon 8L: 5'-GAT GTT CTC TCT GAT TAT GAT GCC-3' and Exon 8R: 5'-CAG ATG ATC TTG CGT ATT ATA ACT GG-3'. The duplication breakpoint was identified by direct sequencing of the ExoSAP (*USB*, Staufen, Germany) purified long distance PCR product applying a primer walking strategy. Sequencing was done employing BigDye Terminator Chemistry 1.1 (*Applied Biosystems*) and products separated on an ABI 3100 DNA Sequencer. Segregation analysis was done applying a duplication specific PCR assay with oligonucleotides: Dupl-Ex7-9-L: 5'-TTC TTG ACA GGT TTG ATA TGG AGA-3' and Dupl-Ex7-9-R: 5'-AAT TAA TGC ATG TCA GTG TCA CCT-3'. In this case PCR was performed using standard Taq polymerase and a short extension time of not more than 30 sec. Numbering of exons and introns was based on *OPA1 *transcript variant 1 (NM_015560).

### Real-Time PCR

Real Time PCR was performed using cDNA generated from total fibroblast RNA with the following oligonucleotides: RT-Ex6-7-f: 5'-AAG AAC TTC TGC ACA CTC AGT TGA A-3' & RT-Ex6-7-r: 5'-TTC TAA TCG TTC CAA GAT TCT CTG ATA C-3'; RT-Ex7-8-f: 5'-GGC ATT CAT CAT AGA AAG CTT AAG AA-3' & RT-Ex7-8-r: 5'-AAG AAC TTC AGA ATA CAT GTC AAT CAA AG-3'; RT-Ex9-7-f: 5'-GGA GAT GAT GAC ACG TTC TCC A-3' & RT-Ex9-7-r: 5'-TTC CAA GAT TCT CTG ATA CTT CAA CTT AA-3'. Assays were carried out with the Power SYBR Green PCR Master Mix on a real time 7500 PCR instrument (*Applied Biosystems*). Relative copy numbers were calculated applying the ΔΔCt method with GAPDH as reference. The pyrosequencing assay has been described in details before [38].

### Amplification of mtDNA

A 12.5 kb mtDNA fragment covering most of the coding region was amplified using TaKaRa LA Taq reagents (*TAKARA BIO INC.*, Shiga, Japan) under recommended cycling PCR conditions and applying the following primer pair: MIT-LR1F: 5'-ACA ACC CTT CGC TGA CGC CAT A-3'; MIT-LR2R: 5'-GGT GGT ACC CAA ATC TGC TTC C-3'.

### Quantitative westernblot analysis

For quantitative Westernblot analysis 15 μg total protein solubilized in RIPA was separated on NuPAGE 4-12% Bis-Tris Gels (*Invitrogen*). Blots were processed for immunodecoration with antibodies against OPA1 (*BD-Transduction*, LA, CA, USA), Tom40 (a kind gift of D. Rapaport, Tuebingen), CoxIV (*Abcam*, Cambridge, MA, USA), ATPase β (*BD-Transduction*), Hsp60 (*Stressgen*, Victoria, Canada) or actin (*Chemicon*, Temecula, CA, USA) applying the ECL chemiluminescence system (*Pierce*, Rockford, IL, USA). According bands were quantified using ImageJ http://rsb.info.nih.gov/ij/. All protein levels were normalized to actin levels as loading control.

## Competing interests

The authors declare that they have no competing interests.

## Authors' contributions

NF performed all genetic analyses including real time experiments; he assisted in the study design, interpreted the data and wrote the paper. SS contributed to the cDNA analysis. YK extracted patients' and control fibroblasts propagated them and provided antibodies. BLK performed all ophthalmological examinations. CA and GA did the field study and provided patients' DNA samples. EZ critically read through the paper. BW critically read through the paper. MVA designed the study, performed all western blot analyses as well as the mitochondrial network analyses, he interpreted the data and wrote the paper. All authors read and approved the final manuscript.
